# Tailored plasmon-induced transparency in attenuated total reflection response in a metal–insulator–metal structure

**DOI:** 10.1038/s41598-017-17847-4

**Published:** 2017-12-19

**Authors:** Kouki Matsunaga, Yusuke Hirai, Yoichiro Neo, Takahiro Matsumoto, Makoto Tomita

**Affiliations:** 1 Department of Physics, Faculty of Science, Shizuoka University, 836, Ohya, Suruga, Shizuoka, 422-8529 Japan; 2 Research Institute of Electronics, Shizuoka University, 3-5-1 Johoku, Naka, Hamamatsu, Shizuoka, 432-8011 Japan; 3 Graduate School of Design and Architecture, Nagoya City University, 2-1-10 Kita-Chikusa, Chikusa, Nagoya, 464-0083 Japan

## Abstract

We demonstrated tailored plasmon-induced transparency (PIT) in a metal (Au)–insulator (SiO_2_)–metal (Ag) (MIM) structure, where the Fano interference between the MIM waveguide mode and the surface plasmon polariton (SPP) resonance mode induced a transparency window in an otherwise opaque wavenumber (*k*) region. A series of structures with different thicknesses of the Ag layer were prepared and the attenuated total reflection (ATR) response was examined. The height and width of the transparency window, as well as the relevant *k*-domain dispersion, were controlled by adjusting the Ag layer thickness. To confirm the dependency of PIT on Ag layer thickness, we performed numerical calculations to determine the electric field amplitude inside the layers. The steep *k*-domain dispersion in the transparency window is capable of creating a lateral beam shift known as the Goos–Hänchen shift, for optical device and sensor applications. We also discuss the Fano interference profiles in a *ω* − *k* two-dimensional domain on the basis of Akaike information criteria.

## Introduction

Induced transparency is a phenomenon in which Fano interference in coupled physical systems creates a narrow transparent window in an otherwise opaque spectral region. Induced transparency has been realized in various systems, such as those found in atomic systems^1–7^, micro resonators^8–10^, meta-materials^11,12^, plasmons^13–15^, and optical mechanical systems^16–18^ in various wavelength regions^19,20^. The relevant steep frequency dispersion in the transparency window produces tunable slow light. This technique has advanced to the point of stopping light pulses completely^3–5^. In realizing induced transparency, the following elements are necessary: a low-Q broad resonance coupled to an incident light field, high-Q resonances to induce the transparency, and coupling between the two resonances. A Λ-shaped, three-level atomic system is the original example of electromagnetically induced transparency (EIT). The ground state and an excited state are coupled by an incident probe field. This transition acts as a low-Q resonance. The system also has a dark state with a slow decay rate. The transition between this state and the ground state acts as a high-Q resonance. An additional laser beam is injected to couple the excited state with the dark state, leading to the destructive quantum interference that cancels the absorption^1^. Similarly, in coupled resonator induced transparency (CRIT), two optical resonators act as low- and high- Q-resonances, and coupling is introduced through the evanescent fields, such that the destructive interference of the two optical pathways cancel the absorption^8–10^. In all systems, a unique and significant feature is that the height of the transparency, as well as the width of the transparent window, is controllable through the adjustment of the coupling strength.

So far, most of the experiments on induced transparency were performed in the frequency (*ω*) domain. Recently, plasmon-induced transparency (PIT) was demonstrated in a wavenumber (*k*) domain, using a metal–insulator–metal (MIM) structure in an attenuated total reflection (ATR) spectrum in a Kretschmann configuration^21,22^. In the proposed system, the MIM waveguide (MIMWG) mode acted as the broad low-Q resonance mode. A sharp high-Q resonance mode was provided by a surface plasmon polariton (SPP) resonance mode, and the Fano interference between two modes induced the transparency window in an otherwise opaque wavenumber (*k*) region.

Here, we experimentally demonstrated that the PIT in a MIM structure can be controlled by adjusting the coupling strength between the MIMWG and SPP resonances. A series of samples with different thicknesses of the Ag layer were prepared and the ATR response was examined. The PIT was controllable through the thickness of the Ag layer. To analyze the dependence of PIT on Ag thickness, we performed numerical calculations for the reflection intensity, the reflection phase shift, and the electric field inside the layers, based on the characteristic matrix method. In the transparency window in the PIT, a steep *k*-domain dispersion exists. This dispersion could modulate the phase of the incident wave packet and displace the reflected beam position. Such displacement is known as the Goos–Hänchen (GH) effect^23^. The GH shift in the PIT in the *k*-domain can be recognized as a rigorous counterpart of slow light in the EIT in the *ω*-domain. The GH effect has been investigated with regard to a wide range of systems, including designed metallic structures^24,25^, photonic crystals with surface waves^26^, metamaterials^27^, and tunable graphene-containing structures^28^. This effect is used in applications such as fiber-based optical switches^29^, narrow band filters^30^, and sensors^31,32^. The tunable and large GH shift observable in the PIT of MIM structures is especially interesting for optical device and sensor applications. Our main interest lies in the *k*-domain profiles of the PIT; however, we also discuss the Fano interference profiles in the *ω* − *k* two-dimensional domain, on the basis of Akaike information criteria.

## Results

### Experiments on the ATR response of a MIM multi-layer structure

**Figure 1:**
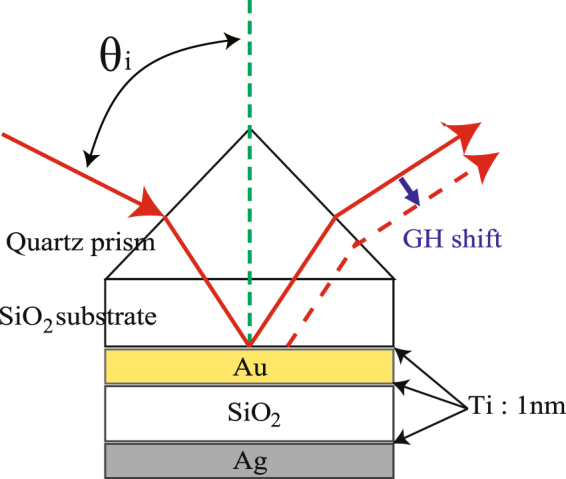
Schematic illustration of the metal–insulator–metal (MIM) (Au/SiO_2_/Ag) structure. The red line represents the incident and reflected beams. The dotted red line represents the Goos–Hänchen (GH) shift observable in the plasmon-induced transparency (PIT) of MIM structures.

Figure 1 shows a schematic diagram of the metal–insulator–metal (MIM) (Au/SiO_2_/Ag) structure used in experiments. Before preparing samples for demonstration of tailored PIT, we performed simulations using the characteristic matrix method^33^. The first layer was Au (thickness, *d*_1_: 40 nm). The thickness of the Au layer was designed to optimize the coupling between the incident light and the MIMWG. The optimal value was identified as the thickness at which the reflection minimum was zero. The second layer was SiO_2_ (thickness *d*_2_: 196 nm). The SiO_2_ layer sustained the MIMWG. The thickness of this layer was designed so that the lowest transverse magnetic (TM) mode in the MIMWG appeared at the same incident angle as the SPP resonance at the Ag–air interface at the incident wavelength of 632.8 nm. The third layer was Ag. We prepared six samples with different Ag layer thicknesses (*d*_3_: 137, 117, 87, 77, 67, and 47 nm for samples 1, 2, 3, 4, 5, and 6, respectively). We deposited thin Ti layers between the metal and insulator layers as buffer layers to form uniform Au, Ag, and SiO_2_ layers.

**Figure 2:**
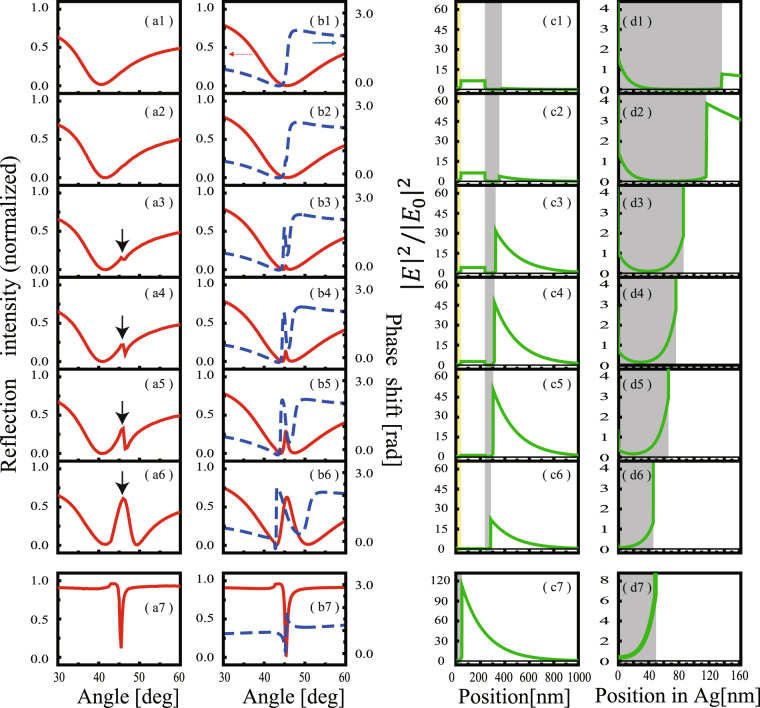
(**a**) Experimental observations of normalized reflection intensity as a function of incident angle *θ*_*i*_ with p-polarization in the MIM structures for samples of different Ag layer thicknesses (**a1**–**a6**) and a single layer of the Ag structure (**a7**). The Ag layer thickness was (**a1**) *d*_3_ = 137, (**a2**) 117, (**a3**) 87, (**a4**) 77, (**a5**) 67, and (**a6**) 47 nm (corresponding to samples 1, 2, 3, 4, 5, and 6, respectively). The Ag layer thickness was 50 nm in (**a7**). (**b**) The solid red and dotted blue lines are calculations for the normalized reflection intensity and reflection phase shift, respectively, corresponding to experiments shown in column (**a**). (**c**) Solid green lines are calculations of the electric field profile inside the MIM structure (**c1**–**c6**), as well as in the single layer of the Ag structure (**c7**) generated with the incident laser beam at *θ*_*i*_ = 45.3°. Downward arrows in (**a**) indicate the transparency window. The yellow and gray regions in (**c**) represent the Au and Ag layers, respectively. (**d**) Expanded plots of the electric field profile in the Ag layer.

**Figure 3:**
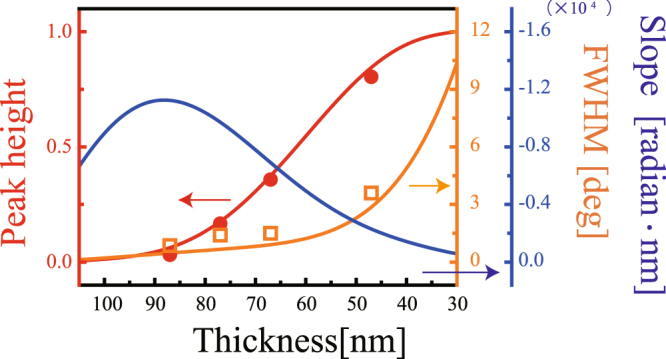
Solid circles and open squares show experimental data for the peak height and full width-half maximum (FWHM) of the PIT in the MIM structure, respectively. Solid red and orange lines are calculated curves for the height and width. The solid blue line is the calculated curve for the slope of the *k*-domain dispersion.

Figure 2(a) shows the experimental results of the reflection intensity as a function of the angle of the incident beam, *θ*_*i*_ observed with p-polarization for samples with different Ag layer thicknesses. Figure 2(a1) shows the spectrum of the sample with an Ag layer thickness of 137 nm (sample 1). A single broad reflection dip appeared at *θ*_*i*_ = 45°. The bandwidth was 27° and the minimum reflectivity was *T*_min_ = 0.01. This broad dip corresponds to the low-Q broad resonance. Figure 2(a2–a6) show the reflection spectra for samples with thinner Ag layers (samples 2–6). As the thickness of Ag layer decreased, a narrow reflection peak grew up in the otherwise opaque angular region in the ATR response. This peak corresponds to the high-Q sharp resonance. Figure 3 summarizes the height and the width of the transparency window as a function of the Ag layer thickness. The height asymptotically approached 1 and the width increased monotonically as the thickness decreased. We refer to this peak as the transparency window and will discuss it in detail in the next section.

### Simulation

The experimental results shown in Fig. 2(a) suggest that the transparency window is induced by the Ag layer. To elucidate the mechanism of the reflection peak, we calculated the reflection intensity based on the transfer matrix method^33^. The red lines in Fig. 2(b) show the calculated curves of the reflection intensity as a function of incident angle *θ*_*i*_. The calculation well reproduced the experimental observations. The width of the transparency window became broad as the thickness of the Ag layer decreased. Figure 2(c) shows the calculated electric field profile inside the MIM structure as a function of position *z*. The z-axis is taken to be perpendicular to the prism (substrate)–Au interface, and the origin is taken at the interface. The first Au layer was located at 0 nm < z < 40 nm [yellow region in Fig. 2(c)], the SiO_2_ layer at 40 nm < z < 236 nm, and the Ag layer at 236 nm < z < 236 + *d*_3_ nm [gray region in Fig. 2(c)]. The negative region of z corresponds to the prism (substrate), where the incident and reflected beams propagated from the left- and right-hand side, respectively. The vertical scale of Fig. 2(c) is normalized with respect to the intensity of the incident beam. Figure 2(c1) shows the electric field profile in the sample with *d*_3_ = 137 nm (sample 1) generated with the incident laser beam at *θ*_*i*_ = 45.3°, which was the resonance angle of the SPP mode expected at the Ag–air interface. In this case, the electric field localized in the SiO_2_ layer, suggesting that the broad reflection dip is associated with the waveguide mode in the SiO_2_ layer. In a MIM structure, the coupling between two SPPs at the two insulator–metal interfaces (Au-SiO_2_ and SiO_2_-Ag) generate symmetric and antisymmetric SPP modes, which are TM modes^21,34^. These modes are plasmonic and couple with the p-polarized incident light. The broad resonance of this mode in the *k* domain indicates that the relevant propagation length along the layer is short. The MIM structure also supports transverse electric (TE) modes, which are essentially photonic modes. These modes couple with s-polarized incident light and do not appear in our spectra. Figure 2(c2–c6) show the electric field profiles inside the MIM structure for thinner Ag layer samples (sample 2–6) generated at incident angle *θ*_*i*_ = 45.3°. As the thickness of the Ag layer decreased, the evanescent field relevant to the MIMWG penetrated the Ag layer and reached the opposite Ag–air boundary, and an electric field amplitude at the Ag–air boundary appeared. This electric field, *E*_*SPP*_, decayed exponentially in both sides of Ag–air interface as an evanescent wave and was attributed to the SPP at the Ag–air interface. Simultaneously, the reflection peaks in Fig. 2(b2–b6) grew, and the electric field inside the SiO_2_ layer, ${E}_{Si{O}_{2}}$, decreased with the thickness of the Ag layer [Fig. 2(c2–c6)]. This electric field distribution inside the SiO_2_ layer is a characteristic feature in the Fano resonance between the broad MIMWG mode and the SPP mode with a higher Q-factor. The incident field excites the SPP through the MIMGW resonance, and the SPP re-emits the phase-shifted electric field. The incident and re-emitted light interfere destructively within the SiO_2_ layer and makes the ${E}_{Si{O}_{2}}$ very small. As the electric field in the SiO_2_ layer is small, the energy absorption in the MIM WG mode is small. Simultaneously, the re-emitted light generates the reflection peak. The observed peak can, therefore, be understood as a PIT as a result of the destructive interference between the two optical pathways: one path passes the SPP and the other path bypasses the SPP; the strength of PIT is thus controllable via the Ag layer thickness. For comparison, Fig. 2(a7,b7 and c7) show experimental observations of normalized reflection intensity, as well as calculations of the electric field profile inside a single Ag layer structure. In the single layer of Ag, the SPP was directly excited through the coupling between the incident light field and the evanescent field relevant to the SPP field.

In Fig. 2(a4,a5), the experimental profiles tend to disagree with the calculations, especially after 45°. This disagreement could be attributed to the slight mismatch between the resonance angles of the MIMFW and SPP resonances in our experiments. As the thickness of the Ag layer was further decreased below 47 nm, and thus the coupling strength was further increased, the refection profile developed distinct double dips.

The blue lines in Fig. 2(b) show calculated curves for the *k*-domain dispersion, *φ*(*k*). As the Ag layer became thinner, the slope of the phase initially steepened with an increase in the height of the transparency window. As the coupling strength increased further and the width of the induced transparency window increased, the slope became less steep (Fig. 3, blue line). The dispersion in the transparency window modulated the phase of the incident wave packet (beam profile) and could displace the reflected beam position. Such a displacement in the reflected optical beam position is referred to as the GH effect^23^. Figure 1 schematically illustrates the GH shift in the PIT in the MIM structure. Consider an incident beam with a finite beam width, Δ*k*_*laser*_ < Δ*k*_*PIT*_, whereΔ*k*_*laser*_ and Δ*k*_*PIT*_ are *k*-domain bandwidths of the incident laser beam and PIT, respectively. In this case, the spatial beam profiles are decomposed into *k* components, and the different *k* components experience different reflection phase shifts due to the *k*-domain dispersion. Specifically, the first-order derivative is responsible for the beam position shift, *D*, *D* = −(*λ*/2*π*)∂*φ*(*θ*)/∂*θ*. The SPP resonance occurs only in a p-polarized incident beam; hence, the GH shift could be observed using the beam profile that occurs in s-polarization as the reference profile^35^.

Induced transparencies have been realized in a wide variety of systems. However, most research investigated induced transparencies in the *ω*-domain. In traditional induced transparency in the *ω*-domain, the width of the frequency window can be controlled by adjusting the coupling strength. The window can be tailored to have an ultra-narrow width. Such a narrow window is accompanied by steep frequency dispersions, which can be used for large displacement of the temporal position of pulses, i.e., slow light^2–5,8^. Temporal pulses are decomposed into *ω*-components, and the different *ω*-components experience different transmission phase shifts, displacing the pulse position in the time domain. The group delay is described by *τ*_*g*_ = ∂*φ*(*ω*)/∂*ω*, where *φ*(*ω*) is the transmitted phase shift^36–38^. In the present case, the transparency window is created in the *k*-domain, and the beam can be reflected by the system with a large, positive GH shift without significant attenuation, amplification, or beam profile deformation. In this context, the GH shift in the PIT in the *k*-domain can be recognized as a rigorous counterpart of slow light in the EIT in the *ω*-domain.

**Figure 4:**
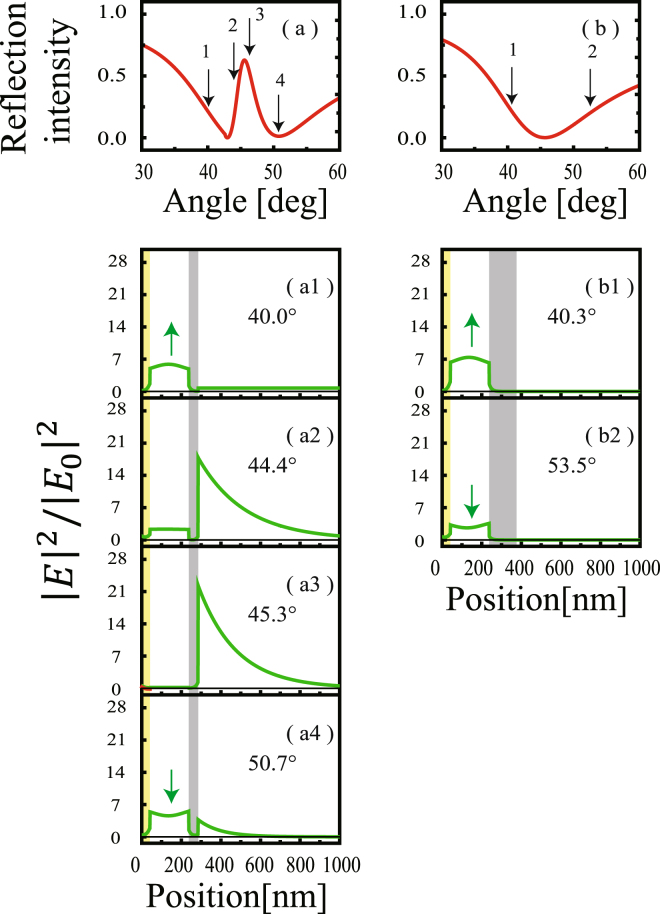
(**a**,**b**) Calculation of the normalized reflection intensity as a function of incident angle *θ*_*i*_, in p-polarization in the MIM structures of samples with Ag layer thicknesses (**a**) *d*_3_ = 47 and (**b**) 137 nm. (a1–a4) Calculation of the electric field distribution inside the MIM structure formed at the incident angle of (a1) *θ*_*i*_ = 40.0°, (a2) 44.4°, (a3) 45.3°, and (a4) 50.7° [indicated as 1, 2, 3, and 4 in (**a**)] in samples of Ag layer thickness *d*_3_ = 47 nm. (b1,b2) Similar calculations for the electric field formed at the incident angle (b1) *θ*_*i*_ = 40.3° and (b2) 53.5° [indicated as 1 and 2 in (**b**)] in a sample with *d*_3_ = 137 nm. The yellow and gray regions represent the Au and Ag layers, respectively.

We further examined the electric field profiles in the MIM structure generated by a beam with different incident angles. Figure 4(a,b) show the field profiles in samples of *d*_3_ = 47 and 137 nm (samples 6 and 1), respectively. Figure 4(a3) is the field profile generated under the same conditions shown for Fig. 2(c6). As discussed for the results shown in Fig. 2, the electric field became strongly localized at the Ag–air interface and the field inside the SiO_2_ layer, ${E}_{Si{O}_{2}}$ was very small. At the lower and higher angles (*θ*_*i*_ = 44.4° and 50.7°), ${E}_{Si{O}_{2}}$ was about the same strength as *E*_*SPP*_ (Fig. 4(a2,a4)). At these angles, the incident beam still partially excited the SPP mode. As the incident angle was further detuned from the SPP resonance angle, the electric field remained only inside the SiO_2_ layer and could not excite the SPP as the phase matching was no longer possible (Fig. 4(a1)). In Fig. 4(a1), the electric field outside the Ag layer (236 + *d*_3_ nm < z) was constant, which suggests that the electric field was a propagation mode and not localized at the Ag–air interface. Additionally, the characteristics of the electric field inside the SiO_2_ layer depended on the incident angle. At higher angles [Fig. 4(a4)], the electric field within the SiO_2_ layer curved downward along the *z*-axis and had a minimum value at the center of the SiO_2_ layer (denoted by the downward arrow). This mode profile is explained by the plasmon fields decaying exponentially from the interfaces Au-SiO_2_ and SiO_2_-Ag layers toward the center region of the SiO_2_ layer. In contrast, in the lower angular region [Fig. 4(a1)], the field profile inside the SiO_2_ layer curved upward along the *z*-axis and had maximum value at the center of the SiO_2_ layer (denoted by the upward arrow). This indicates that the mode is more photonic rather than plasmonic.

Figure 4(b) shows the field profiles in the sample with *d*_3_ = 137 nm (sample 1). In this sample, the electric field existed only inside the SiO_2_ layer, as the SPP at the Ag–air interface was decoupled by the thick Ag layer. The field profiles in the SiO_2_ layer generated by the beam of the lower and higher angular sides (40.3° and 53.5°, respectively) exhibited similar tendencies as the sample with *d*_3_ = 47 nm (Fig. 4(a)). In the lower angular region, MIMWG is more photonic, and in the higher angular region, MIMWG is more plasmonic.

## Discussion

**Figure 5:**
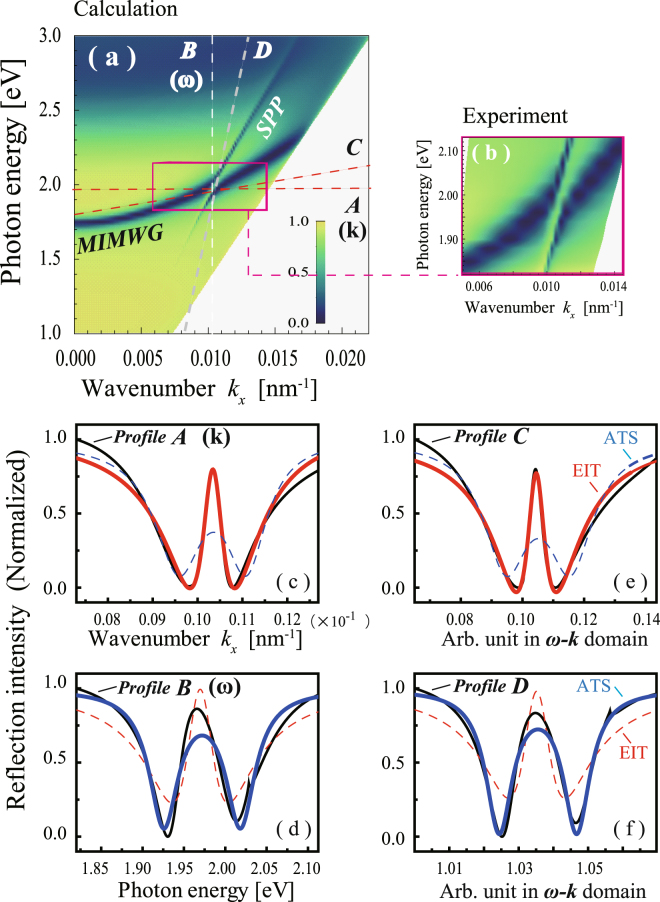
Two-dimensional mapping of the refection intensity in the *ω* − *k* domain of the attenuated total reflection (ATR) spectrum in the Kretschmann configuration of the MIM structure in p polarization (*d*_1_ = 37, *d*_2_ = 191, *d*_3_ = 47 nm). (**a**) Calculated curve and (**b**) experimental observation. (**c**,**d**) Profiles of the refection intensity along the *k*- and *ω*-axes, respectively. (**e**,**f**) Profiles of the refection intensity along other lines (C and D) as indicated in (**a**). The red and blue curves are fitted curves based on Eqs (1) and (2), respectively.

While our interest here lies in the *k*-domain PIT as shown in Fig. 2, the Fano interference that leads to PIT takes place in a *ω* − *k* domain. We can also discuss the Fano profile in the *ω*-domain. Furthermore, we may consider profiles along any directions in the *ω* − *k* domain. Figure 5(a,b) show two-dimensional mappings of the reflection intensity in the *ω* − *k* domain in the ATR spectrum of the MIM structure in p polarization. Figure 5(a,b) show the calculated and experimental results, respectively; two traces of MIMWG and SPP modes are shown, represented by the blue (low reflection) regions. At 2.0 eV, the two traces interfere destructively exhibiting anti-crossing behavior^34^. The black line in Fig. 5(c) shows the profile of the reflection intensity along the *k*-axis (profile A), which is essentially the same as in Fig. 2(a). The black line in Fig. 5(d) shows the profile of the reflection intensity along the *ω*-axis (profile B). The black lines in Fig. 5(e,f) are profiles of the reflection intensity along other lines (profiles C and D) indicated in Fig. 5(a).

Recently, there has been intense discussion of two similar but different effects that both yield transparency in an absorption profile in the presence of a coupling field^39–42^. One is EIT, which arises from Fano interference between two paths and occurs even at very low control intensity. The other effect is Autler Townes splitting (ATS), which appears as the dressed states in the excited level in the presence of a strong coupling field. Because the differences between EIT and ATS are important in applications such as slow light, optical storage, and quantum information processing, different configurations of three-level atomic systems that feature EIT and/or ATS have been studied in detail^39^. Along with the studies based on their physical mechanism, a phenomenological method based on Akaike information criterion (AIC) was introduced. It was proposed that AIC can be used to discriminate between EIT and ATS models simply by looking at the experimentally obtained spectra without prior information on the system^10,40–42^. The method based on AIC was successfully applied to experimental data in cold cesium atoms^41^ and coupled resonators^10^.

We introduced AIC and analyzed the profiles along the different directions in the *ω* − *k* domain. The stationary input–output characteristics of coupled resonators were analyzed^8,10^. When resonators were chosen such that their Q values differed significantly and the coupling strength was weak, the spectral structure was expressed as the sum of two Lorentz profiles with narrow and broad line widths, respectively, centered at zero detuning frequency. This indicates that the system is in the EIT regime. When resonators were chosen such that their Q values were very similar, the spectral structure comprised two individual Lorentz lines having different center frequencies with an equal line width. This indicates that the system is in the ATS regime^10,40–42^. Profiles for the EIT and ATS models can be written, respectively, as

1 $${I}_{EIT}(x)=\frac{{C}_{1}}{1+{x}^{2}/{\gamma }_{a}^{2}}-\frac{{C}_{2}}{1+{x}^{2}/{\gamma }_{b}^{2}}$$

2 $${I}_{ATS}(x)=\frac{{C}_{3}}{1+{(x-\delta )}^{2}/{\gamma }_{0}^{2}}+\frac{{C}_{4}}{1+{(x+\delta )}^{2}/{\gamma }_{0}^{2}}$$

where *C*_1_, *C*_2_, *C*_3_, and *C*_4_ are the amplitude of the Lorentzian function, *γ*_*a*_, *γ*_*b*_, and *γ*_0_ are the widths, and *δ* is detuning. Equation (1) represents the EIT model, and Eq. (2) describes the ATS model. The red and blue curves in Fig. 5(c–f) are fitted curves using Eqs (1) and (2), respectively. The AIC weights exhibit a binary behavior close to 0 or 1 (nearly 0 or 100% likelihood), and there is an abrupt transition from the EIT to the ATS model. For the *k* profile (profile A), AIC_EIT_ (*k*) = 1 − AIC_ATS_ (*k*) ~ 1 and AIC_ATS_ (*k*) ~ 0; therefore, the *k* profile is closer to EIT than ATS. On the other hand, for the *ω* profile (profile B), AIC_EIT_ (*ω*) ~ 0 and AIC_ATS_ (*ω*) = 1 − AIC_EIT_ (*ω*) ~ 1; therefore, the profile is more ATS than EIT. Further, for the profiles shown in Fig. 5(e,f), profiles C and D have the highest and lowest AIC_EIT_ weight, respectively, among the profiles obtained in the *ω* − *k* domain of Fig. 5(a); therefore, profiles C and D are the most typical EIT and ATS profiles, respectively. The shape of the *k*- and *ω*-profiles depend on the parameters of the resonances, such as the bandwidths of MIMWG and SPP, as well as the intersection angle of the two modes in the *ω* − *k* domain. For example, when the bandwidth of MIMWG is broad and that of SPP is narrow, the *k*-profile appears as an EIT type and the *ω*-profile appears as an ATS type.

In Fig. 5, the fitting of the EIT model (red lines) tends to disagree with the experimental results at the edge of the profile, and the ATS model (blue lines) fails at the refection peak. These discrepancies may be attributed to the simplification used in our analysis. Although in real systems the profiles include material complexity, we assumed the profiles exhibited a Lorentz-shaped function, as in Eqs (1) and (2). The discrimination of an EIT-type profile from an ATS-type profile based on AIC may be a convenient tool for judgment of the profiles. The underlying physics in our case is, however, a single effect, i.e., Fano interference between the MIMWG mode and SPP resonances in the *ω* − *k* domain.

## Summary

We demonstrated that the height and width of the transparency window of PIT in the MIM structure can be tailored through adjustment of the Ag layer thickness. In contrast to traditional induced transmission in the *ω*-domain, the transparency window discussed here was realized in the *k*-domain. The GH shift in PIT in the *k*-domain can be recognized as a rigorous counterpart of slow light in EIT in the *ω*-domain. The present system could preserve the sensitivity of SPP sensors, because the final layer sustains SPP and the evanescent field emerges from the final layer. The tunable, giant GH shifts observed in the tunable PIT in MIM structures show great potential for various optical devices and high-sensitivity sensor applications, such as fiber-based optical switches, narrow band filters, and bio-sensors. The observed PIT profile in the *k*-domain can be understood as the intercept profile of the Fano interference between MIMWG and SPP modes in the *ω* − *k* two-dimensional space, where the *ω*-profile is closer to ATS than EIT.

## Method

MIM samples were fabricated on quartz substrates (25 mm × 25 mm × 1 mm) using an electron cyclotron resonance sputtering method. The substrates were attached on a right-angle quartz prism (25 mm × 25 mm × 25 mm) using an index mating oil. The reflected beam intensity was monitored using a photodiode as a function of the incident beam angle in a Kretschmann configuration.

For the measurement of the *k* profiles in the ATR response [Fig. 2(a)], a He-Ne laser was used as the incident light source. For the mapping of Fano interference in the *ω* − *k* two-dimensional domain [Fig. 5(b)], we used a halogen lamp as the incident light source. The collimated light beam from the lamp was passed through a monochromator, polarizer, and 1/2 wavelength plate. The beam had a bandwidth *δλ* = 10 nm in wavelength and *δθ* = 0.5° in angular distribution. The angular ATR response was measured using the collimated beam in 10-nm step increments; from this, a *ω* − *k* two-dimensional space mapping was constructed.
